# Supplementary material for: OpenMM 7: Rapid development of high performance algorithms for molecular dynamics
Source: PLoS Comput Biol. 2017 Jul 26;13(7):e1005659. doi: 10.1371/journal.pcbi.1005659 (PMC5549999; doi:10.1371/journal.pcbi.1005659)
Supplement: S1 Source Code — (ZIP) [file pcbi.1005659.s002.zip › OpenMM-7.0.1-Source/docs/api-python/generated/simtk.openmm.app.pdbxfile.PDBxFile.html]

PDBxFile — OpenMM 7.0.1.dev-5e86c4f documentation


# PDBxFile¶

*class* `simtk.openmm.app.pdbxfile.``PDBxFile`(*file*)¶
:   PDBxFile parses a PDBx/mmCIF file and constructs a Topology and a set of atom positions from it.

    `__init__`(*file*)¶
    :   Load a PDBx/mmCIF file.

        The atom positions and Topology can be retrieved by calling
        getPositions() and getTopology().

        |  |  |
        | --- | --- |
        | Parameters: | **file** (*string*) – the name of the file to load. Alternatively you can pass an open file object. |

    Methods

    |  |  |
    | --- | --- |
    | `__init__`(file) | Load a PDBx/mmCIF file. |
    | `getNumFrames`() | Get the number of frames stored in the file. |
    | `getPositions`([asNumpy, frame]) | Get the atomic positions. |
    | `getTopology`() | Get the Topology of the model. |
    | `writeFile`(topology, positions[, file, ...]) | Write a PDBx/mmCIF file containing a single model. |
    | `writeHeader`(topology[, file, entry]) | Write out the header for a PDBx/mmCIF file. |
    | `writeModel`(topology, positions[, file, ...]) | Write out a model to a PDBx/mmCIF file. |

    `__delattr__`¶
    :   x.\_\_delattr\_\_(‘name’) <==> del x.name

    `__format__`()¶
    :   default object formatter

    `__getattribute__`¶
    :   x.\_\_getattribute\_\_(‘name’) <==> x.name

    `__hash__`¶

    `__reduce__`()¶
    :   helper for pickle

    `__reduce_ex__`()¶
    :   helper for pickle

    `__repr__`¶

    `__setattr__`¶
    :   x.\_\_setattr\_\_(‘name’, value) <==> x.name = value

    `__sizeof__`() → int¶
    :   size of object in memory, in bytes

    `__str__`¶

    `getNumFrames`()¶
    :   Get the number of frames stored in the file.

    `getPositions`(*asNumpy=False*, *frame=0*)¶
    :   Get the atomic positions.

        |  |  |
        | --- | --- |
        | Parameters: | - **asNumpy** (*bool=False*) – if true, the values are returned as a numpy array instead of a list   of Vec3s - **frame** (*int=0*) – the index of the frame for which to get positions |

    `getTopology`()¶
    :   Get the Topology of the model.

    *static* `writeFile`(*topology*, *positions*, *file=<open file '<stdout>'*, *mode 'w'>*, *keepIds=False*, *entry=None*)¶
    :   Write a PDBx/mmCIF file containing a single model.

        |  |  |
        | --- | --- |
        | Parameters: | - **topology** (*Topology*) – The Topology defining the model to write - **positions** (*list*) – The list of atomic positions to write - **file** (*file=stdout*) – A file to write to - **keepIds** (*bool=False*) – If True, keep the residue and chain IDs specified in the Topology   rather than generating new ones. Warning: It is up to the caller to   make sure these are valid IDs that satisfy the requirements of the   PDBx/mmCIF format. Otherwise, the output file will be invalid. - **entry** (*str=None*) – The entry ID to assign to the CIF file |

    *static* `writeHeader`(*topology*, *file=<open file '<stdout>'*, *mode 'w'>*, *entry=None*)¶
    :   Write out the header for a PDBx/mmCIF file.

        |  |  |
        | --- | --- |
        | Parameters: | - **topology** (*Topology*) – The Topology defining the molecular system being written - **file** (*file=stdout*) – A file to write the file to - **entry** (*str=None*) – The entry ID to assign to the CIF file |

    *static* `writeModel`(*topology*, *positions*, *file=<open file '<stdout>'*, *mode 'w'>*, *modelIndex=1*, *keepIds=False*)¶
    :   Write out a model to a PDBx/mmCIF file.

        |  |  |
        | --- | --- |
        | Parameters: | - **topology** (*Topology*) – The Topology defining the model to write - **positions** (*list*) – The list of atomic positions to write - **file** (*file=stdout*) – A file to write the model to - **modelIndex** (*int=1*) – The model number of this frame - **keepIds** (*bool=False*) – If True, keep the residue and chain IDs specified in the Topology   rather than generating new ones. Warning: It is up to the caller to   make sure these are valid IDs that satisfy the requirements of the   PDBx/mmCIF format. Otherwise, the output file will be invalid. |

High performance molecular simulation on GPUs


### Navigation

- Application Layer
  - Loaders and Setup
  - Representation and Manipulation
  - Simulation
  - Reporting Output
  - Extras
- Library Layer

©2015, Stanford University and the Authors.
|
Powered by Sphinx 1.3.1
& Alabaster 0.7.7
|
Page source
